# Spatial distribution of early life stages of fish along horizontal and vertical gradients in the canary current large marine ecosystem

**DOI:** 10.1093/plankt/fbag030

**Published:** 2026-05-20

**Authors:** Omaima Mouiret, Hinde Abdelouahab, Maik Tiedemann, Yassine Goliat, Omar Ettahiri, Tarik Baibai, Ahmed Errhif, Stamatina Isari

**Affiliations:** Laboratory of Health, Environment and Biotechnology, Department of Biology, Faculty of Sciences Ain-Chock, Hassan II University, Km 8 Route d'El Jadida, B.P. 5366, Casablanca 20100, Casablanca-Settat, Morocco; Laboratory of Marine Plankton Ecology, Department of Oceanography, National Institute of Fisheries Research (INRH), 2 Boulevard Sidi Abderrahmane, Casablanca 20250, Casablanca-Settat, Morocco; Laboratory of Marine Plankton Ecology, Department of Oceanography, National Institute of Fisheries Research (INRH), 2 Boulevard Sidi Abderrahmane, Casablanca 20250, Casablanca-Settat, Morocco; Institute of Marine Research (IMR), Sustainable Development, Nordnesgaten 50, Bergen 5005, Vestland, Norway; Laboratory of Marine Plankton Ecology, Department of Oceanography, National Institute of Fisheries Research (INRH), 2 Boulevard Sidi Abderrahmane, Casablanca 20250, Casablanca-Settat, Morocco; Laboratory of Marine Plankton Ecology, Department of Oceanography, National Institute of Fisheries Research (INRH), 2 Boulevard Sidi Abderrahmane, Casablanca 20250, Casablanca-Settat, Morocco; Laboratory of Marine Plankton Ecology, Department of Oceanography, National Institute of Fisheries Research (INRH), 2 Boulevard Sidi Abderrahmane, Casablanca 20250, Casablanca-Settat, Morocco; Laboratory of Health, Environment and Biotechnology, Department of Biology, Faculty of Sciences Ain-Chock, Hassan II University, Km 8 Route d'El Jadida, B.P. 5366, Casablanca 20100, Casablanca-Settat, Morocco; Institute of Marine Research (IMR), Plankton Research Group, Nordnesgaten 50, Bergen 5005, Vestland, Norway

**Keywords:** eastern boundary upwelling systems, northwest Africa, fish larvae, diel vertical migration, assemblage structure, *Sardina pilchardus; Cyclothone*

## Abstract

The Canary Current Large Marine Ecosystem (CCLME) is among the most productive upwelling systems globally, supporting high fish biomass and biodiversity. We examined horizontal patterns of early fish stages along a 241-nautical-mile transect and vertical distributions down to 800 m at four of 11 stations, within the strong permanent upwelling area of the CCLME in December 2019. An inshore–offshore transition of the larval assemblages was observed, shifting from dominance of European pilchard (*Sardina pilchardus*) within the upwelling domain to an offshore assemblage dominated by mesopelagic families such as Myctophidae, Phosichthyidae, and Gonostomatidae. Mesoscale activity near the 3000 m isobath significantly influenced larval distribution patterns. A distinct vertical structuring was evident, with transforming stages of mesopelagic taxa contributing increasingly toward deeper layers. The gonostomatid *Cyclothone* prevailed between 400 and 800 m, showing no evidence of diel vertical migration, unlike other mesopelagic fish species. Size distributions of *Cyclothone* revealed a clear ontogenetic vertical pattern, with larger individuals occurring at greater depths, while transforming stages comprised a substantial proportion of total sampled net biomass within the deeper strata (up to 34.9%). These findings are discussed in the context of the ecology of this genus and its importance to the carbon flow in the mesopelagic zone.

## INTRODUCTION

Eastern Boundary Upwelling Systems (EBUS) rank among the most productive marine ecosystems globally, sustaining rich planktonic communities and supporting major fisheries (Fréon *et al.,* 2009; Lachkar and Gruber, 2013). The Canary Current Large Marine Ecosystem (CCLME) is the largest EBUS worldwide (43°–8°N) (Benazzouz *et al.,* 2014). The northwest African part of the CCLME extends from the Strait of Gibraltar (36°N) to Guinea-Bissau (8°N) and is characterized by intense coastal upwelling, high primary productivity, and strong mesoscale activity (Arístegui *et al.,* 2009; Chavez and Messié, 2009; Kämpf and Chapman, 2016; Sambe *et al.,* 2016). A key biogeographic feature is Cape Blanc (21°N), which separates the quasi-permanent upwelling regime of the northern sector influenced by North Atlantic Central Water (NACW) from the more seasonal regime to the south under the influence of South Atlantic Central Water (Arístegui *et al.,* 2006, 2009; Benazzouz *et al.,* 2014). This latitudinal transition has been recognized as a major ecological boundary within the CCLME for both zooplanktonic (e.g. Weikert, 1982; Berraho *et al.,* 2015; Goliat *et al.,* 2025) and fish abundances (e.g. Hamann *et al.,* 1981; Tiedemann *et al.,* 2018; Diogoul *et al.,* 2021).

North of Cape Blanc (21°N), the upwelling is permanent or quasi-permanent and strongly modulated by mesoscale processes such as filaments and eddies (Barton *et al.,* 1998; Navarro-Pérez and Barton, 1998; Arístegui *et al.,* 2009). While filaments transport nutrient-rich water and early fish life stages offshore mesoscale eddies that remain near the coast enhance larval retention (Barton *et al.,* 1998; Hernández-León *et al.,* 2007; Brochier *et al.,* 2008). This region sustains important and intensive fisheries activity (Kifani *et al.,* 2008), particularly of small pelagic species such as European anchovy, European pilchard, and sardinellas (Lakhnigue *et al.,* 2019; Sarre *et al.,* 2024). These species’ early life stages have been the primary focus of numerous ichthyoplankton studies along the Northwest African coast north of Cape Blanc (Ettahiri *et al.,* 2003, 2012; Berraho *et al.,* 2005, 2012; Bécognée *et al.,* 2009; Abdelouahab *et al.,* 2016, 2017, 2021). Around the Canary Islands, extensive ichthyoplankton studies have additionally been conducted on the influence of mesoscale processes on larval transport, retention, and community structure (e.g. Rodríguez *et al.,* 2004, 2006; Bécognée *et al.,* 2009; Moyano and Hernández-León, 2011; Moyano *et al.,* 2014).

Vertical distribution is also a key aspect of larval fish ecology. Ontogenetic shifts in depth, including shallow diel vertical migrations at larval stages and deeper distributions transforming stages have adaptive roles in feeding, predator avoidance and retention near spawning areas (Fortier and Leggett, 1983; Garrido *et al.,* 2009; Irisson *et al.,* 2010; Rodríguez *et al.,* 2015; Olivar *et al.,* 2018; Loutrage *et al.,* 2024). These behaviors influence dispersal and survival but also affect trophic interactions and biomass fluxes in the water column (Olivar *et al.,* 2018; Hernández-León *et al.,* 2020; Sarmiento-Lezcano *et al.,* 2022). Despite this importance, vertical structuring of ichthyoplankton in the CCLME has been less frequently documented, and most studies have mainly examined patterns down to 200 m, where early stages are typically concentrated (e.g. Moyano *et al.,* 2014; Abdelouahab *et al.,* 2016; Olivar *et al.,* 2016). The distribution of larvae and transforming to adult stages of fish in the deeper mesopelagic and bathypelagic layers has only scarcely been studied in the CCLME (i.e. Dove *et al.,* 2021) and across the Atlantic (e.g. Olivar *et al.,* 2018). However, the mesopelagic zone represents a key component of the ocean’s biological carbon pump (Cavan *et al.,* 2019). Through their diel vertical migrations, mesopelagic fishes and micronekton actively transport organic carbon from surface productive layers to depth, where it is respired and remineralized (Davison *et al.,* 2013). Recent studies have emphasized that this active carbon flux can be comparable to or even surpass the passive sinking of particles (e.g. Hernández-León *et al.,* 2020; Sarmiento-Lezcano *et al.,* 2022), highlighting the major contribution of mesopelagic organisms to oceanic carbon cycling. More information on these scarcely sampled layers is needed.

The present study investigates the assemblages of the early and transforming to adult stages of fish along a 241-nautical-mile inshore–offshore transect off Northwest Africa. By combining horizontal (shelf to oceanic) and vertical sampling (surface to 800 m, at a subset of stations) gradients, we aimed at (i) characterizing patterns in the assemblage structure across environmental and trophic gradients, and (ii) provide one of the first assessments of depth-related biomass partitioning for the early stages of fish in the mesopelagic zone, based on plankton-net collections. This approach advances understanding of survival strategies of larval and transforming stages, the connectivity between coastal and oceanic habitats, and the role of early life stages of fish in plankton-mediated ecosystem processes in the CCLME.

## MATERALS AND METHOD

### Sampling design and sample processing

**Fig. 1:**
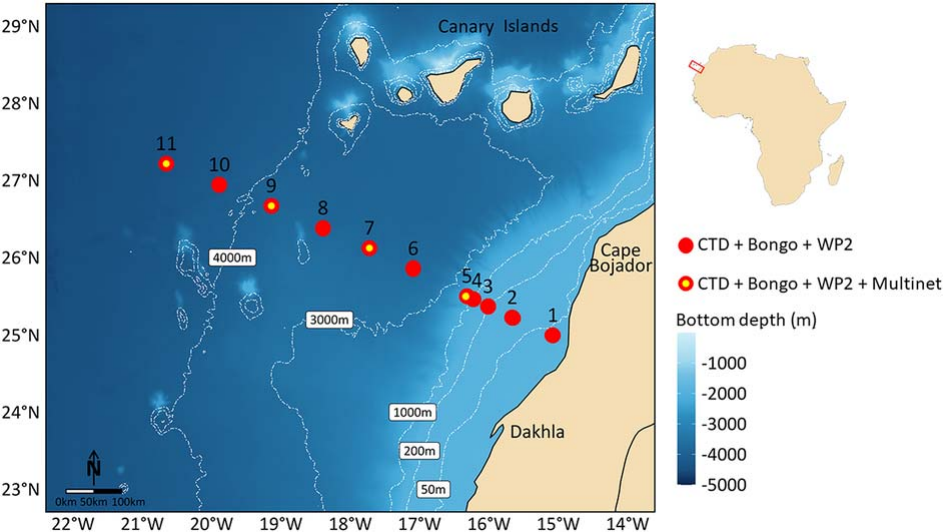
Map showing the geographical distribution of sampling stations using three gears (Bongo/WP2: stations 6 and 10 night sampling, other stations day; Multinet: day sampling on station 5 only, rest of stations both day and night). Bathymetric background illustrates bottom depth. Dashed white contours correspond to the isobaths of 50, 200, 1000, 3000, and 4000 m.

The survey was conducted in winter 2019 (6–14 December) off the Northwest African coast onboard the R/V “*Dr. Fridtjof Nansen*”. Sampling followed a transect oriented perpendicular to the shoreline south of Cape Bojador extending from approximately 24°59′N, 15°05′W to 26°41′N, 19°08′W (Fig. 1). This transect crossed a pronounced environmental gradient from productive inshore coastal waters to the oligotrophic oceanic waters of the Canary Basin, covering depths from ~ 41 m to the 4500 m isobath. Sampling was conducted at 11 stations distributed along this transect (Fig. 1) within a single 24-h period to account for potential diel variability.

Ichthyoplankton sampling was conducted using a Bongo net (Hydrobios 438 750; 60 cm mouth diameter, 405 μm mesh size) equipped with a mechanical flowmeter (Hydrobios 438 110). The net was deployed in double oblique tows to a maximum depth of 200 m, or 10 m above sea floor at stations shallower than 200 m; only two stations (i.e. 6 and 10) were sampled at night. Tow depth was monitored using a Scanmar Depth Sensor attached to the net. At four stations of the transect (i.e. stations 5, 7, 9, and 11; see Fig. 1), depth stratified sampling was additionally carried out using a Multinet Mammoth (Hydrobios 438 163; 1 m^2^ mouth opening, 300 μm mesh size). Two electronic mechanical flowmeters were used: one measured the internal flow of filtered water while the other monitored the external flow relevant for assessing potential clogging. The flowmeter data indicated that filtering performance was optimal, with no signs of clogging or reduced throughput. For both Bongo net and Multinet, the vessel maintained a towing speed of 2–3 knots. Five depth strata were consecutively sampled with the Multinet using single oblique tows at each station (i.e. 800–600 m, 600–400 m, 400–200 m, 200–100 m and 100 m–surface); sampling depth was measured in real time by an integrated pressure sensor on the Multinet system. Multinet sampling was conducted during both day and night conditions at three stations (i.e. stations 7, 9 and 11), while one station (i.e. station 5) was sampled only during the day due to adverse weather. At the final station of the transect (i.e. station 11), the net for the 0–100 m layer (night haul, net N5) was torn, and no sample was obtained for that stratum.

Fish larvae were sorted onboard from fresh Bongo (one net) and Multinet samples, counted, and staged. Horizontal patterns were studied from larvae collected with the Bongo net, and included from preflexion to postflexion larvae. Incidental collections of juvenile or adult fish were not included. Vertical distribution patterns were analyzed separately for larval and transforming/juvenile stages (i.e. individuals in the process of completion of photophore development and attainment of juvenile-adult characters (Kendall *et al.,* 1984)). In the case of the *Cyclothone* species, the term “juveniles to adult” has been used, since it was not possible to distinguish between juvenile and adult stages.

Taxonomic identification was carried out to the lowest possible level based on morphological characteristics and standard taxonomic keys (Fahay, 2007; Olivar and Fortuño Alós, 1991; Rodríguez *et al.,* 2017; Rodríguez, 2023). Counts were standardized to individuals per m^2^ for the Bongo net and per 1000 m^3^ for Multinet. The dominant taxa (i.e. the European pilchard *S. pilchardus* and the gonostomatid *Cyclothone* spp.) from each gear type (Bongo and Multinet, respectively) were further processed measuring total body length on frozen specimens. For biomass estimation, *Cyclothone* life stages from Multinet samples were pooled separately by sample on pre-weighed aluminum trays and oven-dried at ~ 60°C for 24 h to estimate dry weight (expressed as mg per 1000 m^3^). The same drying procedure was applied separately to the remaining specimens (larval and transforming/juvenile fish stages), after identification to the lowest possible taxonomic level.

Mesozooplankton dry weight in the upper 0–200 m was used as proxy of productivity based on vertical tows of a WP2 net (56 cm mouth diameter, mesh size of 64 μm) equipped with a mechanical flowmeter (Hydrobios 438 115). Tows extended from 200 m (or 5 m above the seabed at shallower stations) to the surface and the net was retrieved at a speed of ∼0.5 m s^−1^; only two stations (i.e. 6 and 10) were sampled at night. Samples were halved with a Motoda plankton splitter (Motoda, 1959). One half was used for size-fractionated dry weight measurements using sequential sieving through 2000, 1000, and 180 μm meshes (Skjoldal *et al.,* 2013) to provide an indication of zooplankton biomass. Each size fraction was oven-dried at ∼60°C for 24 h in pre-weighed aluminum trays on board, and dry weight was expressed as g m^−2^. The same procedure was applied to the Multinet samples (after removal of fish larvae) to measure mesozooplankton dry weight across depth strata. The second half of WP2 samples were fixed in a 4% borax-buffered formaldehyde solution and later used to count European pilchard (*S. pilchardus*) eggs.

At each plankton station, temperature, salinity, dissolved oxygen and fluorescence were measured with a Sea-Bird 911plus CTD profiler equipped with a WET Labs ECO-AFL fluorometer mounted on a rosette with 12 Niskin bottles (10 L) for depth stratified water sampling. Chlorophyll-a (Chl-a) concentration was estimated from fluorescence measurements. The fluorometer readings were calibrated using Chl-a values obtained from water samples collected at various depths with Niskin bottles. Salinity was cross validated onboard using a Portasal Salinometer (Model 8410A). Dissolved oxygen concentrations were determined using Winkler titration (Grasshoff *et al.,* 1983).

### Remote-sensing data and CTD-derived visualizations

Environmental parameters, including Sea Surface Temperature (SST), Sea Surface Salinity (SSS), and geostrophic currents derived from Sea Surface Height (SSH), were retrieved from the Copernicus Marine Service (https://marine.copernicus.eu/). Daily data were averaged over an eight-day period to generate contour maps illustrating the general oceanographic conditions during the survey. Data extraction and visualization were performed in R (Version 4.5.0; R Core Team, 2025) using R-Studio (Version 4.5.0; R Studio Team, 2025) and associated R packages “ncdf4” (Pierce, 2010) and “terra” (Hijmans, 2020) to highlight mesoscale structures and circulation patterns. Vertical sections of environmental variables from CTD casts, including temperature, salinity, dissolved oxygen, and chlorophyll-a at different distances from the coast (i.e. inshore stations: 1–2; shelf-break: 3; offshore stations: 4–11), were generated using Ocean Data View (ODV v5.7.0; Schlitzer, 2024).

### Data analyses

All statistical analyses were conducted and related plots were produced, in R using R-Studio and the R packages “vegan” (Oksanen *et al.,* 2022) and “ggplot2” (Wickham, 2016).

Variations in the relative abundance and size structure of *S. pilchardus* larvae were analyzed by grouping individuals into two size classes: < 7 mm (<4 days old) and ≥ 7 mm (4–7 days old), based on mean growth rate of ~ 0.6 mm day^−1^ (Ettahiri, 1996). The 7 mm threshold corresponds to the appearance of the dorsal fin that marks an important transition from low to enhanced swimming abilities (Ettahiri, 1996; Berraho, 2007). Differences in larval abundance and size distribution among stations were assessed using the non-parametric Kruskal-Wallis test.

Variation in the structure of larval fish assemblages (larval stages only) in the upper 200 m across the transect was investigated using the Bongo net data. Abundances of identified taxa (larvae m^−2^) were square-root transformed to reduce the influence of dominant species and a Bray–Curtis’s dissimilarity matrix was built afterwards. Hierarchical clustering together with non-metric multidimensional scaling (nMDS) (Clarke *et al.,* 2014) were carried out on this matrix using the group-average linkage method to examine the structure of the larval assemblages. Statistical significance (α = 0.05) of the clusters was assessed with the Similarity Profile test (SIMPROF; Clarke et al., 2014) as implemented in the “clustsig” package (Version 1.1). Similarity Percentage analysis (SIMPER) using the “*simper*” function was used to identify the taxa contributing to the 70% similarity of the clustered assemblages. To further explore taxon co-occurrence, only larval taxa contributing more than 2% in at least one sample were retained. Pairwise Bray–Curtis similarities among these taxa were calculated on larval abundances, followed by agglomerative hierarchical clustering using the “pheatmap” package (Kolde, 2019). The taxonomic richness and the Shannon-Wiener index (H′) (Shannon and Weaver, 1949) at the genus level were calculated for each cluster group.

To examine how environmental factors influence larval structure, we fitted selected environmental variables onto the nMDS ordination using the “*envfit*” function from the “vegan” package (Oksanen *et al.,* 2022). The *envfit* procedure relies on permutations to determine which environmental variables best explain the variation in larval distribution. In this approach, the ordination scores from the nMDS are regressed against each environmental factor individually. The significance of these relationships was assessed through 999 permutations and *P*-values were Benjamini–Hochberg corrected. The environmental factors considered included sampling depth as well as mean values of temperature, salinity, chlorophyll-a, and oxygen in the upper 0–30 m layer.

The Weighted Mean Depths (WMD) over the full sampled depth range (0–800 m, using all five strata) were calculated for (i) transforming/juvenile stages of taxa detected during day and night hauls when both were available (i.e. stations 7, 9 and 11); (ii) *Cyclothone* larvae and transforming to adult stages, as follows:


$$WMD={\sum}_{i=1}^n{P}_i{Z}_i$$

Where ${Z}_i$ is the depth of the stratified haul (the center point of each sampled interval), and ${P}_i$ is the proportion of fish at that depth (Fortier and Leggett, 1983). The Diel Vertical Migration (DVM) was quantified as the difference between WMD night and WMD day values *(DVM = WMD*_night_**​***—WMD*_day_). A length–weight relationship for transforming stages of *Cyclothone*  $W={aL}^b$, where *a* is a scaling coefficient and *b* is the allometric exponent.

## RESULTS

### Remote sensing, hydrographic structure, and zooplankton dry weight along the transect

**Fig. 2:**
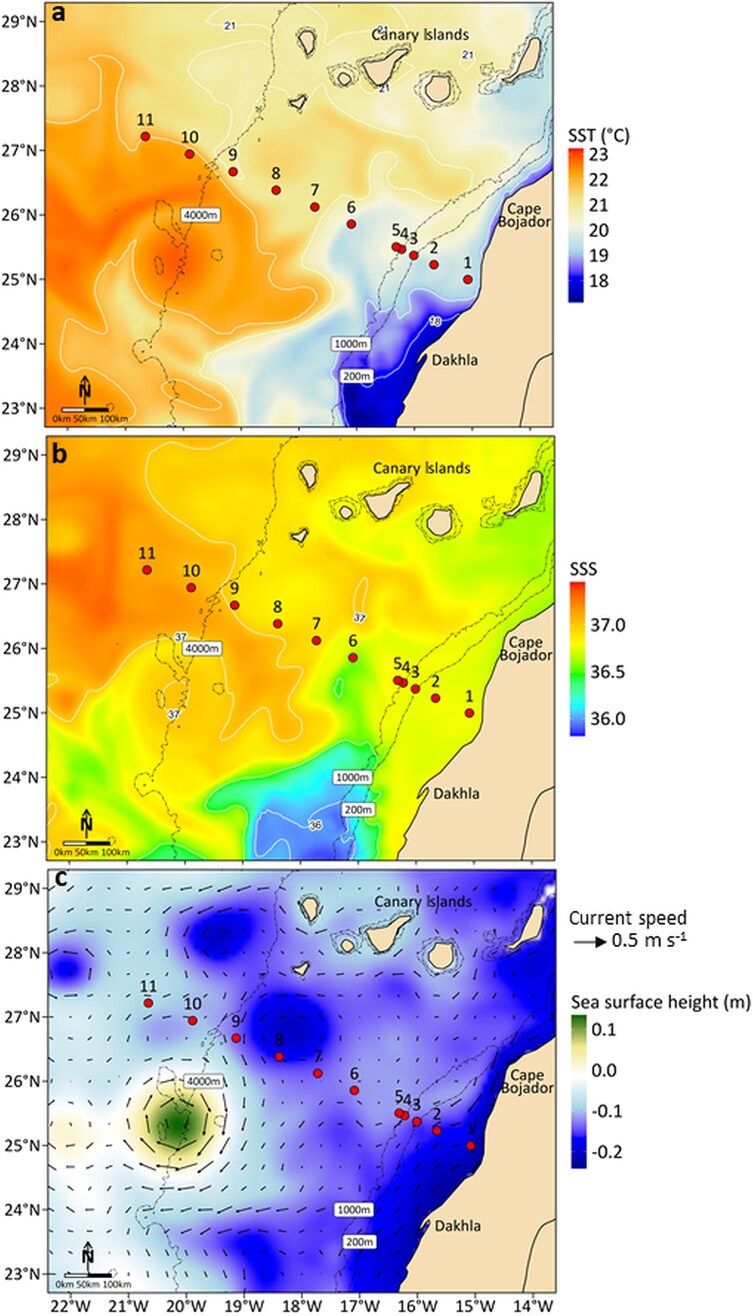
Surface contour maps showing the horizontal distribution across the transect of **(a)** Sea Surface Temperature (SST, °C) with white contour lines representing isotherms, **(b)**Sea Surface Salinity (SSS) with white contour lines representing isohalines, and **(c)**Sea Surface Height (SSH; background color) and geostrophic surface currents (black vectors). Sampling stations are indicated by red dots. Dashed black contours correspond to the isobaths 200 m, 1000 m, and 4000 m.

Satellite observations revealed a gradient in surface conditions from the coastal shelf toward the open ocean (Fig. 2). SST contours (Fig. 2a) indicated an offshore warming with SST increasing from 18–19°C at the inshore and shelf-break stations (1–3) to values exceeding 22°C at offshore stations (6–11). This thermal gradient was accompanied by relatively fresher surface waters (SSS ≈ 36.3–36.4) extending offshore from the Cape Bojador–Dakhla sector and more saline waters (>36.5) to the northwest (Fig. 2b). Additionally, satellite data highlighted the presence of an eddy dipole structure (Fig. 2c), consisting of a cyclonic eddy centered north of stations 8–9 (SST ≈ 21.5°C) and an anticyclonic eddy to the southwest (SST ≈ 23°C).

**Fig. 3:**
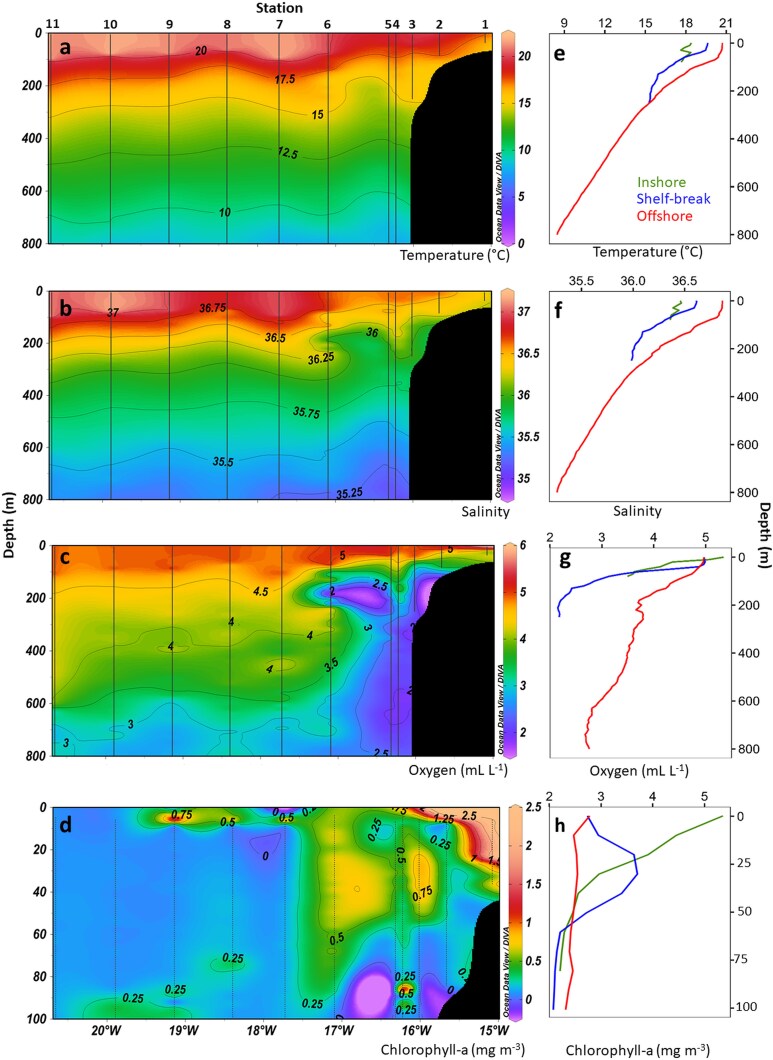
Vertical sections **(a-d)** and profiles (e-h) of the environmental parameters: temperature (°C), salinity, dissolved oxygen (mL L^−1^) and chlorophyll-a (mg m^−3^). CTD cast locations (number of station) are indicated at the top of the sections and by vertical black lines. Vertical profiles represent means at different distances from the coast (i.e. inshore stations: 1–2; shelf-break: 3; offshore stations: 4–11). Chlorophyll-a is shown over a reduced depth range and uses its own depth axis.

Along-transect sections (Fig. 3a–d) showed evidence of coastal upwelling. The 20–17.5°C isotherms shoaled toward the inshore and shelf break, while 36.2–36.6 isohalines converged to form a distinct haline front between stations ~ 4 and 6. Near-surface, Chl-a was elevated in the upper 0–40 m, ranging from 0.8–2.5 mg m^−3^ inshore and at the shelf break. Dissolved oxygen decreased offshore toward a subsurface minimum of ~ 2.5–3.5 mL L^−1^ at 300–600 m. Temperature decreased progressively with depth, and the thermocline deepened offshore (Fig. 3e), indicating higher mixing at the shore with increasing stratification offshore. Salinity was lower inshore and increased offshore, with a deeper halocline beyond the shelf. Chl-a concentrations were enhanced in inshore and shelf-break waters, while oxygen declined gradually with depth following the stratified structure of the water column. The Oxygen Minimum Zone was more developed and pronounced at the inshore stations (OMZ; ~ 2–2.5 mL L^−1^), whereas offshore profiles displayed a more vertically extended OMZ, with slightly higher concentrations (~3 mL L^−1^).

**Fig. 4:**
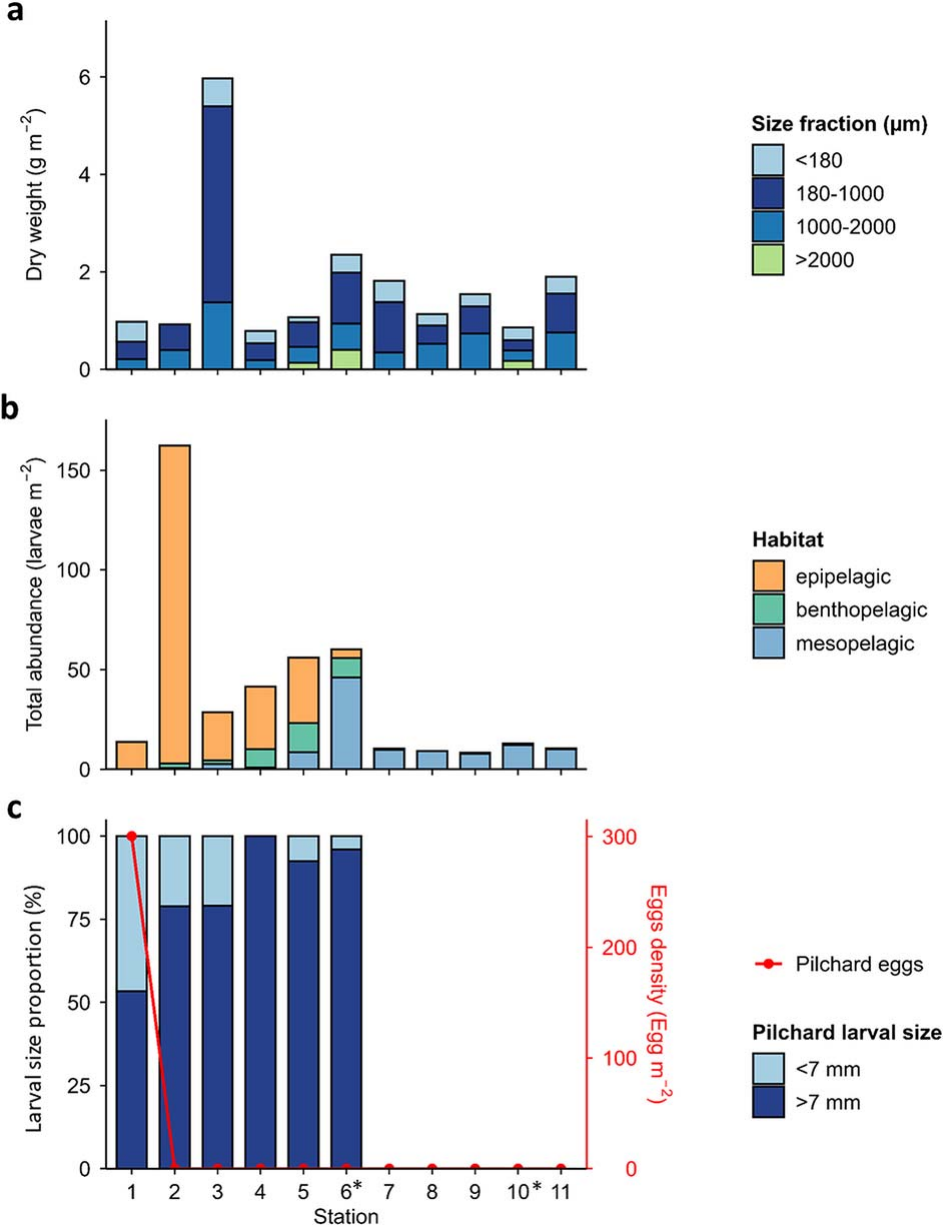
Barplots of **(a)** Mesozooplankton dry weight (mg m^−2^) fractions (<180 μm, 180–1000 μm, 1000–2000 μm, > 2000 μm) sampled with WP2 net; **(b)** Total larval abundance (larvae m^−2^) along the transect (inshore stations: 1–2, shelf-break: 3, offshore stations: 4–11) in the upper 200 m sampled with the Bongo net. Bars are colored by adult habitats (epipelagic, benthopelagic, mesopelagic); and **(c)** Larval size proportion (%) of European pilchard larvae, expressed as the percentage contribution of each size class (based on the standard length (SL): < 7 mm and ≥ 7 mm) to the total larval abundance at each station, sampled with the Bongo net. The red curve represents the density of European pilchard eggs sampled with WP2 net (Egg m^−2^). Stations with asterisk (^*^) symbol are sampled at nighttime.

Mesozooplankton dry weight (Fig. 4a) was relatively uniform across stations, with notable peaks at station 3 (shelf break) and station 6 (offshore). Mean dry weight was 0.95 ± 0.04 g m^−2^ inshore and 1.43 ± 0.56 g m^−2^ offshore, whereas values reached 5.95 g m^−2^ at the shelf break offshore, with no statistically significant differences among bathymetric zones (Kruskal-Wallis test: χ^2^ = 3.41, df = 2, *P* = 0.18). Contributions of different size fractions varied among stations and sampling time with larger fractions most prominent at offshore samples collected at night.

### Structure of larval fish assemblage

#### Horizontal distribution patterns of fish larvae

A total of 1339 fish larvae were sorted from the Bongo net collections comprising 28 families and at least, 28 genera and 61 species (Table I). Total larval fish abundance varied markedly along the transect, ranging from 0.21 to 159.01 larvae m^−2^ (Fig. 4b). The highest abundances were observed inshore (station 2) and at an offshore station (i.e. station 6) located close to the 3000 m isobath (Fig. 4b). A spatial shift in larval fish assemblages was evident across the transect, associated with differences in adult habitat (Fig. S1). *S. pilchardus* was the major epipelagic species along the transect with abundances ranging between 4.06 and 159.01 larvae m^−2^, where its presence was confined to stations 1 to 6. A significant spatial shift in size distribution was evident for *S. pilchardus* across the transect (Kruskal–Wallis’s test, χ^2^ = 83.07, df = 5, *P* < 0.001), with smaller larvae (<7 mm) predominating the most inshore stations (Fig. 4c). Consistent with this inshore dominance, eggs of *S. pilchardus* occurred exclusively at station 1, reaching 300.45 eggs m^−2^ (Fig. 4c).

**Table I:** List of taxa identified in the Bongo net collections along the transect. For each taxon, the adult habitat (epipelagic, mesopelagic, or benthopelagic), the mean larval abundance (Larvae m^−2^ (LA) ± standard deviation (SD)), and the frequency of occurrence (FO%, proportion of stations where present) are provided

Adult habitat	Family	Taxa	LA ± SD	FO (%)
**epipelagic**	Belonidae	Belonidae sp.	0.25 ± 0.08	9.09
	Carangidae	Carangidae sp.	0.68 ± 0.32	27.27
		*Caranx* sp.	0.30 ± 0.09	9.09
	Clupeidae	*Sardina pilchardus*	43.84 ± 46.59	54.55
	Nomeidae	Nomeidae sp.	0.21 ± 0.06	9.09
**benthopelagic**	Anguillidae	Anguillidae sp.	0.21 ± 0.06	9.09
	Blenniidae	Blenniidae sp.	0.65 ± 0.31	18.18
	Bothidae	Bothidae sp.	0.31 ± 0.06	27.27
	Caproidae	*Capros aper*	0.04 ± 1.06	9.09
	Centriscidae	*Macroramphosus* spp.	1.25 ± 0.56	18.18
	Fistularidae	Fistularidae sp.	0.61 ± 0.08	9.09
	Gobiidae	Gobiidae spp.	0.91 ± 0.36	18.18
	Labridae	Labridae sp.	0.25 ± 0.08	9.09
	Serranidae	Serranidae sp.	0.23 ± 0.07	9.09
	Sparidae	Sparidae spp.	1.08 ± 0.54	54.55
	Trachinidae	*Trachinus* sp.	0.49 ± 0.15	9.09
	Trichiuridae	*Lepidopus caudatus*	2.04 ± 1.39	36.36
	Triglidae	Triglidae sp.	0.50 ± 0.15	9.09
**mesopelagic**	Bathylagidae	*Melanolagus bericoides*	8.45 ± 2.55	9.09
	Evermannellidae	*Evermannella* sp.	0.21 ± 0.06	9.09
	Gempylidae	Gempylidae sp.	0.39 ± 0.20	27.27
	Gonostomatidae	*Cyclothone* spp.	0.58 ± 0.40	54.55
		*Diplophos* sp.	0.56 ± 0.17	9.09
		*Scopelarchus* spp.	0.30 ± 0.09	9.09
		*Sigmops elongatus*	0.23 ± 0.07	9.09
	Macrouridae	Macrouridae sp.	1.50 ± 0.45	9.09
	Myctophidae	*Benthosema suborbitale*	0.46 ± 0.24	27.27
		*Bolinichthys indicus*	0.43 ± 0.22	27.27
		*Ceratoscopelus maderensis*	1.17 ± 0.43	18.18
		*Diaphus rafinesquii*	0.21 ± 0.06	9.09
		*Diaphus* spp.	0.90 ± 0.52	45.45
		*Gonichthys cocco*	0.41 ± 0.19	18.18
		*Hygophum bruuni*	0.76 ± 0.23	9.09
		*Hygophum macrochir*	0.75 ± 0.23	9.09
		*Hygophum proximum*	0.65 ± 0.30	27.27
		*Hygophum reinhardtii*	0.45 ± 0.22	27.27
		*Hygophum* spp.	0.69 ± 0.21	9.09
		*Hygophum taaningi*	0.77 ± 0.38	27.27
		*Lampanyctus* spp.	0.63 ± 0.30	45.45
		*Lobianchia* spp.	0.35 ± 0.15	18.18
		Myctophidae sp.	0.76 ± 0.38	27.27
		*Myctophum nitidulum*	0.61 ± 0.18	9.09
		*Myctophum punctatum*	0.21 ± 0.06	9.09
		*Myctophum* spp.	0.23 ± 0.07	9.09
		*Nannobrachium* sp.	0.21 ± 0.06	9.09
	Notosudidae	*Notoscopelus* spp.	1.10 ± 0.52	18.18
		Notosudidae sp.	0.23 ± 0.07	9.09
	Paralepididae	*Lestidiops jayakari*	0.64 ± 0.19	9.09
		Paralepididae sp.	0.58 ± 0.28	18.18
	Phosichthyidae	*Pollichthys mauli*	0.68 ± 0.21	9.09
		*Vinciguerria attenuata*	0.61 ± 0.18	9.09
		*Vinciguerria nimbaria*	0.68 ± 0.36	36.36
		*Vinciguerria poweriae*	0.23 ± 0.07	9.09
		*Vinciguerria* spp.	0.42 ± 0.13	9.09
	Sternoptychidae	*Maurolicus* sp.	0.90 ± 0.78	63.64
		Sternoptychinae sp.	0.48 ± 0.19	18.18
		*Sternoptyx* spp.	0.71 ± 0.34	18.18
	Stomiidae	Astronesthinae spp.	0.42 ± 0.19	18.18
		*Idiacanthus* spp.	0.23 ± 0.10	18.18
		Melanostominae spp.	0.21 ± 0.06	9.09
		Stominae spp.	0.43 ± 0.13	9.09
**Destroyed / Unidentified**			2.61 ± 2.24	63.64

**Fig. 5:**
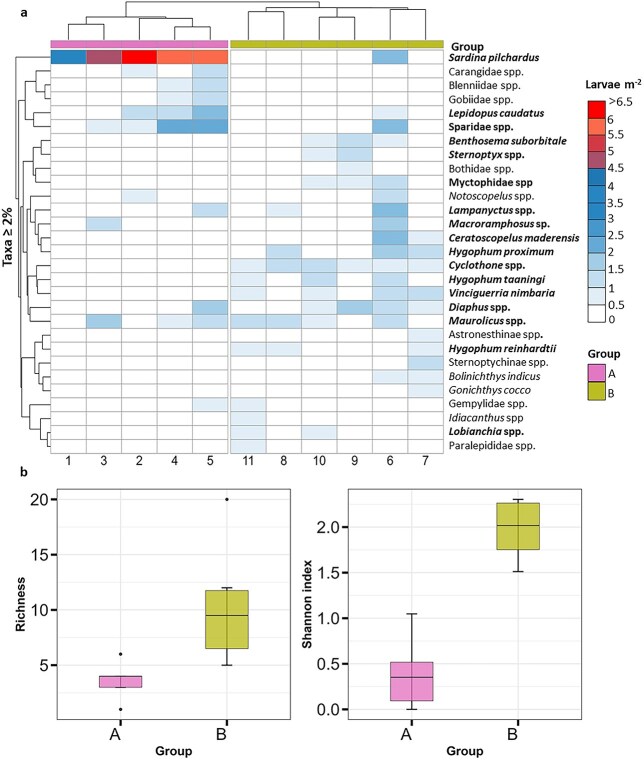
**(a)** Heatmap of square-root transformed larval fish abundances (larvae m^−2^) of taxa contributing to more than 3% (>3% of total abundance) across Bongo net sampling stations. Groups (A and B) identified by the cluster analysis (see Fig. S2 supplementary data) are shown in the color bar. Taxa contributing with > 70% similarity described by the SIMPER analysis (Table S1) are presented with bold font. **(b)** Boxplots of diversity indices between the two stations groups with taxonomic richness (left) and the Shannon diversity (right) based on genus level. The upper and lower quartiles determine the interquartile range of the box plot. The horizontal black line within the box indicates median. Black dots outside the box represent outliers.

**Fig. 6:**
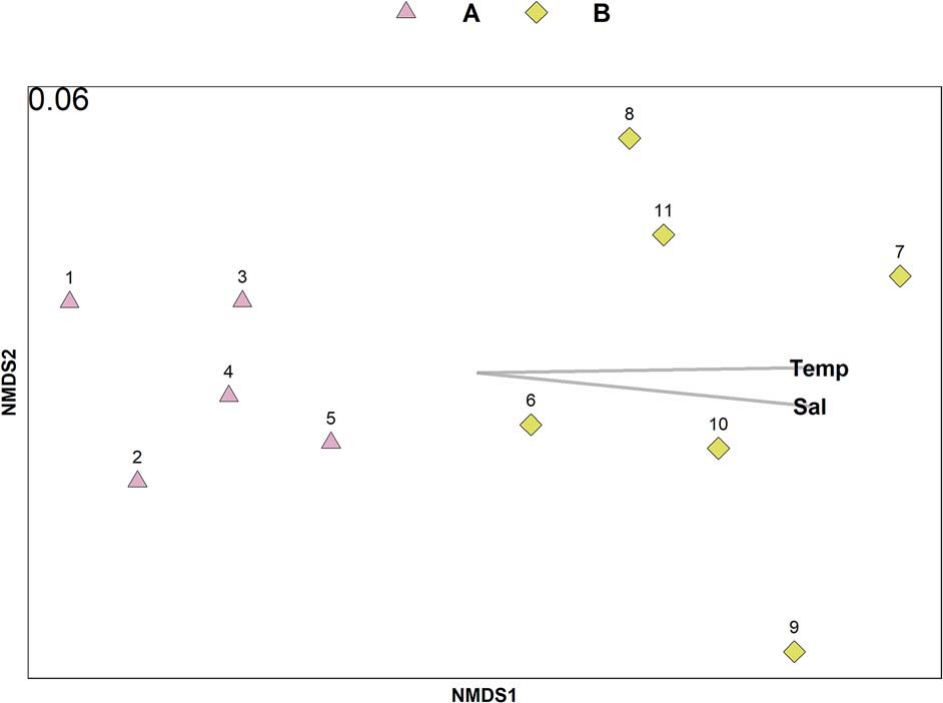
Non-metric multidimensional scaling (nMDS) plot of the larval-fish community based on Bray-Curtis dissimilarities and square-root transformed abundances. Cluster group information based on the cluster analysis (see Fig. S2 supplementary data) has been superimposed. Stress value is shown at the upper left corner. Significantly correlated environmental vectors (*P* < 0.05, identified with *Envfit* function) fitted to the nMDS are shown.

Toward offshore, the contribution of epipelagic taxa progressively declined, accompanied by an increase in larvae of benthopelagic fish taxa (e.g. the trichiurid *Lepidopus caudatus*) as well as an increase in several mesopelagic taxa (Fig. 5b, Fig. S1). Cluster analysis at 79% dissimilarity distinguished two groups of stations (Fig. 5a, Fig. S2), which were also evident in the nMDS ordination (Fig. 6). The five most inshore stations (Group A, stations 1–5) were grouped together and were characterized by the dominance of *S. pilchardus* (Fig. 5a, Table S1) and low taxonomic richness and diversity (Fig. 5b). In contrast, the remaining stations (stations 6–11) formed Group B, which exhibited higher taxonomic richness and diversity, with similarity driven by the contribution of mesopelagic species (Fig. 5a and b, Table S1). Notably, station 6 displayed the highest diversity across the transect and a high *Macroramphosus* spp. abundance (1.25 ± 0.56 larvae m^−2^). Among the mesopelagic taxa, only larvae of the genera *Maurolicus* and *Notoscopelus* had a broader spatial distribution occurring both inshore and at the shelf break at depths of 89 and 255 m respectively, while others such as *Hygophum, Diaphus* and *Lampanyctus* were present at the deepest station (i.e. station 5) of Group A (the inshore cluster). Larvae of all remaining mesopelagic taxa occurred exclusively at stations located over bottom depths greater than 3000 m. Group A was negatively correlated with temperature and salinity (the inshore group) while group B was positively correlated with these parameters (Fig. 6, Table II). The remaining environmental variables, including sampling depth, chlorophyll-a, and oxygen, were not significantly associated with either group.

**Table II:** *Fits of selected environmental vectors to the nMDS ordination of larval fish assemblages. Environmental vectors: mean Temperature (Temp), Salinity (Sal), Dissolved Oxygen (Oxy) and Chlorophyll-a (Chl-a) in the 0–30 m layer and station sampling depth (Depth).* r^2^*: Correlation coefficient and Pr(>r): P-value.* Pr(>r)_BH: p-values adjusted for multiple testing using the Benjamini–Hochberg procedure*. Significant values are indicated in bold, with significance defined as P* < 0.01(^**^)

Variables	nMDS1	nMDS2	***r*** ^***2***^	Pr(>r)	Pr(>r)_BH
Temp	1.000	0.015	0.860	0.002	0.008^**^
Oxy	0.423	−0.897	0.057	0.796	0.796
Sal	0.995	−0.102	0.824	0.003	0.008^**^
Chl-a	−0.982	0.190	0.469	0.088	0.143
Depth	0.965	0.261	0.427	0.051	0.085

#### Vertical distribution patterns of larval, transitional, and adult stages of fish

**Fig. 7:**
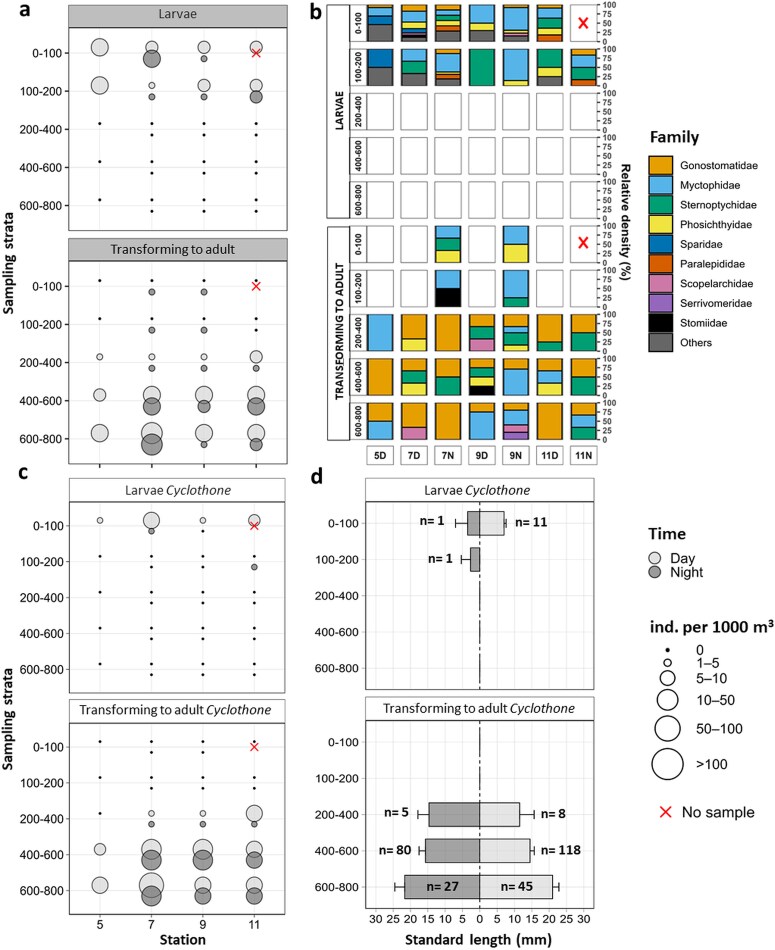
Bubble plots of early-life and transforming stages of **(a)** Total larval and **(c)**  *Cyclothone* densities (ind. per 1000 m^3^) during daytime (light gray) and nighttime (dark gray) across the five sampled depth layers (0–100, 100–200, 200–400, 400–600, 600–800 m) of the water column collected with the Multinet. Relative density (%) plots of **(b)** Larval families of larval and transforming stages at each station and depth layer. White cells denote absence at the corresponding depth and stage. **(d)** Vertical distributions of mean standard length (mm) of *Cyclothone* for transforming and larval stages collected with the Multinet. Standard errors are shown sideways the bars and number of individuals (n) are indicated. Light conditions during sampling are indicated by D (Day) or N (Night) next to the station number. The red crosse symbol denotes strata with no sample.

A total of 579 individuals were collected (236 larvae and 343 transforming to adult stages) in Multinet samples representing 20 families, and at least 26 genera and 55 species, mostly mesopelagic (Table III, Fig. S3). Preflexion to postflexion individuals were concentrated in the upper 0–200 m layer, where approximately 57% of them occurred within the upper layer (0–100 m) under both light conditions. Postflexion larvae were only occasionally observed below 200 m (Fig. 7a), whereas transforming to adult stages peaked deeper (i.e. 400–800 m), reaching densities exceeding 100 ind. per 1000 m^3^. Larval assemblages exhibited a vertical structure (Fig. 7b), with higher richness and diversity in the 0–200 m layer (Fig. S4). Larval stages of mesopelagic species belonging to the families Myctophidae (e.g. *Hygophum hygomii, Hygophum taaningi, Notolychnus valdiviae*), Phosichthyidae (e.g. *Vinciguerria* spp.*, Vinciguerria nimbaria*) and Gonostomatidae (e.g. *Sigmops elongatum*) prevailed in the upper 200 m (Fig. 7b, Table III). Interestingly, although the larvae of the gonostomatid *Cyclothone* occurred in relatively low densities within the upper 200 m (Fig. 7b), its transforming to adult stages were common in deeper strata (400–800 m), ranging from 1.2 to 201.8 ind. per 1000 m^3^ and contributing on average to 41.7% and 49.8% of total fish assemblages in the 400–600 m and 600–800 m strata, respectively. Size distributions of *Cyclothone* revealed a clear ontogenetic vertical pattern (Fig. 7d): larvae (6–8.9 mm) dominated the surface layers, whereas larger individuals (11–25.5 mm) were abundant below 200 m during both day and night. Length-weight relationship for the transforming to adult stages is provided in Fig. S5. No upward migration of transforming to adult *Cyclothone* stages was detected during night sampling (Fig. 7c; Table S2). However, transforming/juvenile stages of several mesopelagic taxa, including myctophids (Myctophidae spp.), phosichthyids (*Vinciguerria* spp.), sternoptychids (*Maurolicus* spp. and *Argyropelecus hemigymnus*) and stomiids were observed in the upper 200 m during the two-night tows (Fig. 7b, Table S2).

**Table III:** List of taxa identified in the Multinet collections along the transect along with their frequency of occurrence (FO%) and the mean relative density (RD%) at each sampling stratum: 0–100 m, 100–200 m, 200–400 m, 400–600 m, and 600–800 m. For each taxon, the adult habitat (epipelagic, mesopelagic, or benthopelagic) and the family are indicated

Stage/Habitat/Family	Taxa	0–100 m				100–200 m				200–400 m				400–600 m				600–800 m			
		RD%		FO%		RD%		FO%		RD%		FO%		RD%		FO%		RD%		FO%	
		Day	Night	Day	Night	Day	Night	Day	Night	Day	Night	Day	Night	Day	Night	Day	Night	Day	Night	Day	Night
**LARVAE**																					
**epipelagic**																					
Carangidae	Carangidae spp.	3.95	-	2.63	4.17	-	-	-	-	-	-	-	-	-	-	-	-	-	-	-	-
**benthopelagic**																					
Anguilidae	Anguillidae spp.	2.63	1.61	5.26	-	-	-	-	3.33	-	-	-	-	-	-	-	-	-	-	-	-
Bathylagidae	*Melanolagus* sp.	-	-	-	4.17	5.88	-	7.69	-	-	-	-	-	-	-	-	-	-	-	-	-
Fistularidae	Fistularidae sp.	-	-	-	-	-	1.45	-	3.33	-	-	-	-	-	-	-	-	-	-	-	-
Scaridae	Scaridae sp.	-	1.61	-	-	-	-	-	-	-	-	-	-	-	-	-	-	-	-	-	-
Scorpaenidae	Scorpaenidae sp.	-	-	-	-	-	1.45	-	-	-	-	-	-	-	-	-	-	-	-	-	-
Serranidae	Serranidae sp.	1.32	-	2.63	-	-	-	-	-	-	-	-	-	-	-	-	-	-	-	-	-
Sparidae	Sparidae spp.	11.82	-	5.26	-	17.68	-	7.69	-	-	-	-	-	-	-	-	-	-	-	-	-
Trichiuridae	*Lepidopus caudatus*	2.63	-	2.63	-	-	-	-	-	-	-	-	-	-	-	-	-	-	-	-	-
**mesopelagic**																					
Gempylidae	Gempylidae spp.	1.32	-	2.63	-	-	2.90	-	3.33	-	-	-	-	-	-	-	-	-	-	-	-
Gonostomatidae	*Cyclothone braueri*	-	1.61	-	4.17	-	-	-	-	-	-	-	-	-	-	-	-	-	-	-	-
	*Cyclothone pallida*	1.32	-	2.63	-	-	-	-	-	-	-	-	-	-	-	-	-	-	-	-	-
	*Cyclothone pseudopallida*	7.89	-	2.63	-	-	-	-	-	-	-	-	-	-	-	-	-	-	-	-	-
	*Cyclothone* spp.	2.63	1.61	5.26	4.17	-	2.90	-	6.67	-	-	-	-	-	-	-	-	-	-	-	-
	*Sigmops elongatum*	-	-	-	-	-	2.90	-	3.33	-	-	-	-	-	-	-	-	-	-	-	-
Myctophidae	*Benthosema* spp.	-	1.61	-	4.17	-	1.45	-	3.33	-	-	-	-	-	-	-	-	-	-	-	-
	*Benthosema suborbitale*	-	-	-	-	-	5.80	-	6.67	-	-	-	-	-	-	-	-	-	-	-	-
	*Ceratoscopelus* spp.	-	6.45	-	4.17	-	-	-	-	-	-	-	-	-	-	-	-	-	-	-	-
	*Diaphus* spp.	6.58	-	7.95	-	-	8.70	-	10.04	-	-	-	-	1.22	1.51	8.33	10.00	2.17	-	9.09	-
	*Hygophum hygomii*	7.89	-	2.63	-	-	-	-	-	-	-	-	-	-	-	-	-	-	-	-	-
	*Hygophum proximum*	-	-	-	-	-	26.06	-	6.67	-	-	-	-	-	-	-	-	-	-	-	-
	*Hygophum* spp.	11.82	25.83	5.26	4.17	-	-	-	-	-	-	-	-	-	-	-	-	-	-	-	-
	*Hygophum taaningi*	5.26	3.23	5.26	4.17	-	-	-	-	-	-	-	-	-	-	-	-	-	-	-	-
	*Lampanyctus* spp.	1.32	1.61	2.63	4.17	-	2.90	-	3.33	-	-	-	-	-	-	-	-	-	-	-	-
	*Lepidophanes* sp.	-	1.61	-	4.17	-	-	-	-	-	-	-	-	-	-	-	-	-	-	-	-
	*Lobianchia spp.*	2.63	-	2.63	-	-	-	-	-	-	-	-	-	-	-	-	-	-	-	-	-
	Myctophidae spp.	-	-	-	-	5.88	-	7.69	-	-	-	-	-	-	-	-	-	4.35	-	9.09	-
	*Myctophum nitidulum*	1.32	-	2.63	-	-	-	-	-	-	-	-	-	-	-	-	-	-	-	-	-
	*Myctophum reinhardtii*	-	-	-	-	-	1.45	-	3.33	-	-	-	-	-	-	-	-	-	-	-	-
	*Notolychnus valdiviae*	-	11.29	-	4.17	-	-	-	-	-	-	-	-	-	-	-	-	-	-	-	-
Notosudidae	Notosudidae spp.	3.95	3.23	5.26	8.30	5.88	-	7.69	-	-	-	-	-	-	-	-	-	-	-	-	-
Paralepididae	Paralepididae spp.	2.63	4.84	2.63	4.17	-	-	-	-	-	5.56	-	9.09	-	-	-	-	-	-	-	-
	*Paralepis elongata*	-	-	-	-	-	4.35	-	3.33	-	-	-	-	-	-	-	-	-	-	-	-
	*Sudis* sp.	-	-	-	-	-	1.45	-	3.33	-	-	-	-	-	-	-	-	-	-	-	-
Phosichthyidae	*Pollichthys mauli*	1.32	-	2.63	-	5.88	-	7.69	-	-	-	-	-	-	-	-	-	-	-	-	-
	*Vinciguerria attenuata*	1.32	-	2.63	-	-	-	-	-	-	-	-	-	-	-	-	-	-	-	-	-
	*Vinciguerria nimbaria*	1.32	4.84	2.63	4.17	-	-	-	-	-	-	-	-	-	-	-	-	-	-	-	-
	*Vinciguerria poweriae*	1.32	-	2.63	-	-	-	-	-	-	-	-	-	-	-	-	-	-	-	-	-
	*Vinciguerria* spp.	5.26	11.29	5.26	4.17	-	7.25	-	6.67	6.67	-	11.11	-	-	-	-	-	-	-	-	-
Scopelarchidae	Scopelarchidae sp.	1.32	-	2.63	-	-	-	-	-	-	-	-	-	-	-	-	-	-	-	-	-
	*Scopelarchus* spp.	-	1.61	-	4.17	-	-	-	-	6.67	-	11.11	-	-	-	-	-	4.35	-	18.19	-
Sternoptychidae	*Argyropelecus hemigymnus*	-	-	-	-	11.76	1.45	15.41	3.33	6.67	11.11	11.11	9.09	-	1.50	-	10.00	-	-	-	-
	Maurolicinae sp.	1.32	-	2.63	-	-	-	-	-	-	-	-	-	-	-	-	-	-	-	-	-
	*Maurolicus* spp.	-	1.61	-	4.17	-	-	-	-	6.67	11.11	11.11	18.19	-	-	-	-	-	-	-	-
	Sternoptychinae spp.	-	1.61	-	4.17	11.76	-	7.69	-	26.65	16.67	11.11	9.09	-	-	-	-	-	-	-	-
	*Sternoptyx* spp.	3.95	-	2.63	-	5.88	4.35	7.69	3.33	-	-	-	-	-	-	-	-	-	5.00	-	12.50
Stomiidae	Astronesthinae sp.	1.32	-	2.63	-	-	-	-	-	-	-	-	-	-	-	-	-	-	-	-	-
	*Idiacanthus* spp.	-	-	-	-	-	1.45	-	3.33	-	-	-	-	-	-	-	-	-	-	-	-
**Unidentified**		1.32	9.68	2.63	8.30	5.88	11.59	7.69	6.67	-	-	-	-	1.22	0.75	8.33	10.00	-	-	-	-
**LATER STAGES**																					
**mespelagic**																					
Gonostomatidae	*Cyclothone acclinidens*	-	-	-	-	11.76	-	7.69	-	-	-	-	-	-	-	-	-	-	-	-	-
	*Cyclothone* spp.	1.32	-	2.63	-	5.88	2.90	7.69	3.33	40.00	38.87	33.34	27.27	90.24	93.23	33.33	30.00	82.62	85.00	36.36	37.50
Myctophidae	*B. suborbitale*	-	-	-	4.17	-	-	-	-	-	-	-	-	-	-	-	-	-	2.50	-	12.50
	*Bolinichthys indicus*	-	1.61	-	4.17	-	2.90	-	3.33	-	-	-	-	-	-	-	-	-	-	-	-
	*Ceratoscopelus warmingii*	-	-	-	-	-	1.45	-	3.33	-	-	-	-	-	-	-	-	-	-	-	-
	*Diaphus* spp.	-	-	-	-	-	-	-	-	-	-	-	-	-	0.76	-	10.00	2.17	-	9.09	-
	*H. taaningi*	-	-	-	-	-	-	-	-	-	5.56	-	9.09	-	-	-	-	-	-	-	-
	*Lampanyctus pusillus*	-	-	-	-	-	-	-	-	-	-	-	-	-	-	-	-	2.17	-	9.09	-
	Myctophidae spp.	-	1.61	-	4.17	-	1.45	-	3.33	6.67	-	11.11	-	-	-	-	-	2.17	-	9.09	-
	*N. valdiviae*	-	-	-	-	-	1.45	-	3.33	-	-	-	-	-	0.75	-	10.00	-	2.50	-	12.50
Phosichthyidae	*V. attenuata*	-	-	-	-	-	-	-	-	-	5.56	-	9.09	1.22	-	8.33	-	-	-	-	-
	*V. poweriae*	-	-	-	-	-	-	-	-	-	-	-	-	1.22	-	8.33	-	-	-	-	-
	*Vinciguerria* spp.	-	-	-	-	-	-	-	-	-	-	-	-	1.22	-	8.33	-	-	-	-	-
Scopelarchidae	*Scopelarchus* spp.	-	-	-	-	-	-	-	-	-	-	-	-	-	-	-	-	-	2.50	-	12.50
Serrivomeridae	*Serrivomer beanii*	-	-	-	-	-	-	-	-	-	-	-	-	-	-	-	-	-	2.50	-	12.50
Sternoptychidae	*A. hemigymnus*	-	-	-	-	-	-	-	-	-	-	-	-	2.44	0.75	16.69	10.00	-	-	-	-
Stomiidae	*Chauliodus danae*	-	-	-	-	-	-	-	-	-	-	-	-	1.22	-	8.33	-	-	-	-	-
**Unidentified**		-	-	-	-	5.88	-	7.69	-	-	5.56	-	9.09	-	0.75	-	10.00	-	-	-	-

**Fig. 8:**
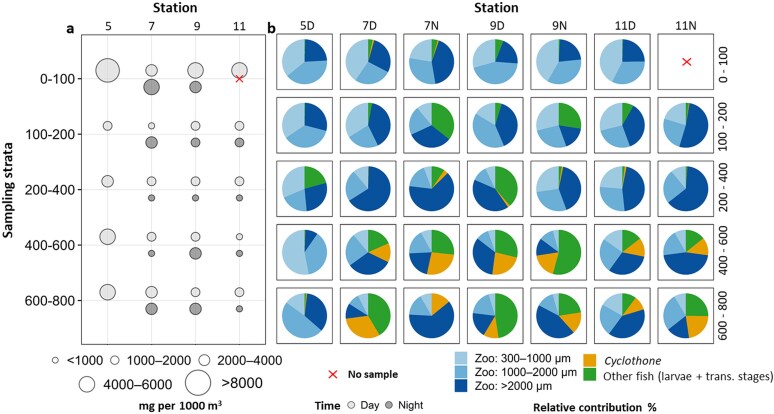
Bubble plots of **(a)** Total dry weight (mg per 1000 m^3^) of mesozooplankton and larval fish and pie plots **(b)** indicating the contribution of bulk mesozooplankton dry weight fractions (300–1000; 1000–2000, and > 2000 μm), *Cyclothone* and other fish collected with the Multinet in the different depth strata (0–100, 100–200, 200–400, 400–600, 600–800 m) along the transect during daytime (D) and nighttime (N).

Station 5, the multinet station closest to the shore, exhibited higher zooplankton biomass across all depth strata compared to the other stations. Overall, the biomass was highest in the upper 0–200 m, with maximum values exceeding 8000 mg per 1000 m^3^ during daytime (Fig. 8a). Below 200 m, biomass declined markedly, although the layer between 400 and 800 m showed some peaks of biomass values ranging between 2232 and 5576 mg per 1000 m^3^ primarily at station 5, with additional elevated values at stations 7 and 9. Size-fractionated biomass of zooplankton, together with the biomass of *Cyclothone* and other fish early life stages, showed a distinct vertical distribution pattern (Fig. 8b): small- and medium-sized zooplankton (300–1000 μm and 1000–2000 μm, respectively) dominated the surface layers, while larger zooplankton (>2000 μm) and *Cyclothone* contributed substantially to the strata below 400 m. At the inshore station, fish biomass (*Cyclothone* and other fish) was almost absent in the two deepest layers with 0.2% at 400–600 m and 0.8% at 600–800 m (Fig. 8b). Larval stages contributed marginally to the total biomass in the upper 200 m from our plankton samples (ranging from 0–1.2% of the biomass), with the highest value (1.2%) recorded in the daytime sample at station 7D in the upper layer (0–100 m), driven mainly by Sparidae spp. (Fig. 8b). Below the epipelagic zone (i.e. 600–800 m), transforming to adult stages of *Cyclothone* accounted for up to 34.9% of the total biomass (station 7D). Other mesopelagic taxa collected in these deeper layers (e.g. myctophids, phosichthyids, scopelarchids; Table III) collectively contributed up to 54.2% of total biomass (station 9 N, 400–600 m; Fig. 8b).

## DISCUSSION

### Inshore-offshore gradient in assemblage structure

A pronounced inshore–offshore gradient in both larval abundance and taxonomic composition was evident along the transect revealing two distinct larval assemblages closely associated with the offshore increase in temperature and salinity. The Northwest African part of the CCLME region where the transect was located (between Cape Bojador and Cape Blanc) is characterized by permanent strong upwelling year-round (Arístegui *et al.,* 2009; Fréon *et al.,* 2009; Benazzouz *et al.,* 2014; Cropper *et al.,* 2014). Accordingly, cooler waters, with high Chl-a and mesozooplankton biomass prevailed inshore, while warmer and more saline conditions characterized the offshore domain. This offshore increase in temperature and salinity reflected the influence of the NACW, which dominates beyond the upwelling front and contributes to the characteristic thermohaline gradient of the Canary Current system (Lázaro *et al.,* 2005; Moyano *et al.,* 2014; Pelegrí and Benazzouz, 2015; Olivar *et al.,* 2016). Interestingly, neither station depth nor Chl-a showed a significant association with the assemblage structure. The lack of a depth effect may partly reflect the clustering of shallow and deep stations within the same group (e.g. Group A), while the absence of a Chl-a signal is likely related to its high spatial variability, probably driven by the hydrological features encountered in the area during the study period. The survey was conducted in early December, coinciding with the known spawning period of European pilchard, *S. pilchardus* (Ettahiri *et al.,* 2003; Bécognée *et al.,* 2009). Since *S. pilchardus* is one of the most common small pelagic fish species in the region, the dominance of its larvae in the inshore assemblage was expected. European pilchard congregates in coastal upwelling zones in the CCLME to feed and spawn (Ettahiri *et al.,* 2003). The upwelling carries nutrient-rich deep water to the surface fueling primary productivity (Kämpf and Chapman, 2016) that is grazed down by zooplankton (Hernández-León *et al.,* 2019a; Goliat *et al.,* 2025), supporting larval and adult fish feeding (Déniz-González *et al.,* 2016). A recent study by Goliat et al. (2025) conducted in the same area, with sampling conducted just weeks prior to our sampling, reported high concentrations of the lipid-rich calanoid species Calanoides natalis (an indicator of upwelling). These findings are consistent with our own observations from the inshore Bongo net samples. Mesozooplankton abundance peaked near the shelf break and not inshore where most spawning was observed, likely reflecting top-down control on mesozooplankton (Goliat *et al.,* 2025). The reduced biomass of the smallest size fraction at the same station may also reflect grazing pressure on early copepod stages (nauplii and early copepodites) exerted by the abundant early-stage *S. pilchardus* larvae or alternatively, a delayed response of the mesozooplankton community to recent shifts at lower trophic levels.

The mean size of larval European pilchards increased progressively offshore until they were no longer present beyond approximately 3000 m bottom depth (i.e. station 6), which agrees with previous findings along the Northwest African coast (Berraho *et al.,* 2012; Abdelouahab *et al.,* 2017). This cross-shelf shift in larval pilchard abundance and size strongly suggests that spawning occurs inshore where eggs and newly hatched larvae are concentrated and that larvae are subsequently advected and dispersed offshore. The high abundance of pilchard eggs encountered at the most coastal station (i.e. station 1) supports this interpretation and aligns with previous studies from the strong upwelling area of the CCLME between Cape Bojador and Cape Blanc (e.g. Ettahiri *et al.,* 2003; Abdelouahab *et al.,* 2021). In contrast, larvae of the benthopelagic species *L. caudatus* increased in abundance with distance from the coast. However, based on our personal observations (data not shown), eggs of this species were found primarily inshore, suggesting offshore advection. Such processes have been widely documented in the CCLME in association with upwelling filaments, both for zooplankton (e.g. Hernández-León *et al.,* 2007; Arístegui *et al.,* 2009) and for fish larvae (Rodríguez *et al.,* 1999, 2004, 2009; Berraho *et al.,* 2005; Moyano and Hernández-León, 2009; Moyano *et al.,* 2014).

The offshore larval assemblages (i.e. station 5 and onward) mostly comprised by common mesopelagic families (e.g. Myctophidae, Gonostomatidae, and Sternoptychidae). This pattern reflects the bathymetric range and reproductive behavior of the adult (e.g. Olivar *et al.,* 1998; Vargas-Yáñez and Sabatés, 2007) and is commonly reported in oceanic waters in Northwestern Africa (Rodríguez *et al.,* 1999, 2006; Moyano and Hernández-León, 2009). Contrasting to the general offshore dominance of mesopelagic taxa, larvae of *Maurolicus* and *Notoscopelus* occurred inshore, which may reflect the pseudo-oceanic/neritic affinity of *Maurolicus* and the relatively shallow distribution of adults near continental slopes (Sobradillo *et al.,* 2019) as well as advection of early stages driven by mesoscale oceanographic processes (i.e. along-slope current interaction with frontal structures) within the Canary Current system (Rodríguez *et al.,* 2004; Moyano *et al.,* 2014). Previous studies conducted in the upper 200 m layer of the water column in offshore waters off Northwest Africa have reported that the myctophid genera *Ceratoscopelus, Hygophum* and *Diaphus* generally dominate the larval assemblage (Rodríguez *et al.,* 1999; Moyano and Hernández-León, 2009, 2011). In our collections, the genus *Hygophum* was particularly dominant. We note that two offshore stations sampled at night were likely influenced by the diel vertical migration of mesopelagic taxa (discussed later) and by increased catchability of postflexion larvae under low light conditions (Somarakis et al., 1998). However, we consider it unlikely that these factors substantially affected the delineation of assemblages.

Our results showed that station 6, although classified within the offshore assemblage, was located in a frontal transition zone between two surface salinity regimes. The location was characterized by high larval abundances, including a marked presence of *Macroramphosus* larvae, a genus known for episodic adult population outbursts along the Northwest African shelf (Brêthes, 1979; Idrissi *et al.,* 2024). The hydrographic structure and the vertical distribution of Chl-a extending down to approximately 80 m depth, suggest the influence of mesoscale circulation and the presence of productive fronts. Such processes are known to enhance biological productivity and play a potential role in larval retention which is considered beneficial for larval survival. This hydrographic setup may also explain the high larval fish diversity at this site as used by several species for reproduction. Previous studies in the region have highlighted the role of eddies south of the Canary Islands in the accumulation of zooplankton biomass (e.g. Hernández-León *et al.,* 2007; Goliat *et al.,* 2025) and the retention of fish larvae (Rodríguez *et al.,* 1999, 2001, Rodríguez *et al.,* 2004; Bécognée *et al.,* 2009; Moyano *et al.,* 2014; Olivar *et al.,* 2016). The anticyclonic eddy was located close to station 8, which exhibited similar abundances of mesopelagic taxa to those at adjacent stations, suggesting a limited influence of the eddy on the local larval assemblage.

### Vertical gradients in assemblage structure and biomass partitioning

Several studies in the CCLME have demonstrated that within the 0–200 m layer the vertical distribution of early fish stages may vary according to taxon-specific preferences based on light conditions, food availability, predator avoidance and developmental stages (e.g. Rodríguez *et al.,* 2006; Moyano and Hernández-León, 2009; Olivar *et al.,* 2016; Abdelouahab *et al.,* 2017). While our study did not resolve the fine-scale patterns within the epipelagic zone addressed by previous works (e.g. Moyano *et al.,* 2014; Olivar *et al.,* 2016), it represents one of the very few ichthyoplankton studies in the Atlantic to extend sampling into deeper strata (down to 800 m), thereby capturing transforming and adult stages (Olivar *et al.,* 2018; Dove *et al.,* 2021).

Our results showed a clear vertical structuring of the larval fish assemblage across the 0–800 m water column, with diversity peaking in the upper 200 m layer where early life stages predominated. Across stations, this layer was dominated by mesopelagic taxa, except at station 5, where we encountered a mixed assemblage comprised of mesopelagic, benthopelagic and epipelagic taxa, consistent with previous observations in the region (e.g. Moyano and Hernández-León, 2009; Moyano *et al.,* 2014). In agreement with Olivar *et al.* (2017), early developmental stages were largely confined to the epipelagic zone, while transforming to adult stages became progressively more abundant with depth, indicating an ontogenetic shift in vertical distribution. Since spawning season and larval occurrences for *Cyclothone* spp. in the NE Atlantic and Mediterranean have been reported from spring to autumn (Badcock, 1984; Sabatés *et al.,* 2007; Olivar *et al.,* 2018), the scarcity of larvae of *Cyclothone* species in the upper 200 during the present survey is likely related to the sampling month, out of the spawning period. However, abundances of transforming to adult stages contributed substantially to both structure and biomass of the mesopelagic zone.

The genus *Cyclothone* is widely recognized as the most abundant vertebrate on earth (Nelson *et al.,* 2016) and constitutes a dominant component of meso- and bathypelagic assemblages across the world oceans (e.g. Sutton *et al.,* 2008, 2010; Olivar *et al.,* 2012, 2017). Because conventional midwater trawls often have a relatively large mesh size through which these small, slender fish can be extruded, stratified sampling with large plankton nets, such as that used in this study, has proven to be more effective (e.g. Olivar *et al.,* 2012, 2017; Agersted *et al.,* 2021). This approach has enabled detailed investigations into the ecology of juvenile *Cyclothone* (Olivar *et al.,* 2017; Sarmiento-Lezcano *et al.,* 2022) and provided valuable ground-truth data for refining acoustic-based biomass estimates (Agersted *et al.,* 2021). In our study, transforming to adult stages of *Cyclothone* were identified only to the genus level, without species-level resolution. However, previous studies in the Atlantic, including areas close to our transect, have documented juveniles/adults of several co-occurring *Cyclothone* species as the most dominant components of the assemblage (e.g. *C. braueri, C. livida, C. microdon, C. pallida, and Cyclothone pseudopallida*) (Olivar *et al.,* 2017; Agersted *et al.,* 2021; García-Seoane *et al.,* 2021; Sarmiento-Lezcano *et al.,* 2022). These latter correspond well with at least some of the *Cyclothone* species identified at earlier developmental stages in our Multinet collections.

Most of the transforming to adult stages of *Cyclothone* were concentrated in the intermediate mesopelagic zone between 400 and 800 m depth, consistent with previous observations (e.g. Olivar *et al.,* 2012, 2017; Sarmiento-Lezcano *et al.,* 2022). Body lengths measured in our study were comparable to those reported by Agersted *et al.* (2021) from Multinet tows north of the Canary Islands, where individuals ranged from 12 to 28 mm. Converting our values to individuals per m^2^ for comparison with that study yielded a similar mean abundance across stations (17 ± 9.3 ind. per m^2^). Our densities within the 400–800 m layer across stations and light conditions (26–81 ind. per 1000 m^3^), were similar to those reported for corresponding depth strata in the North East Atlantic (Sarmiento-Lezcano *et al.,* 2022), but substantially lower than those found by Olivar *et al.,* 2017 in the tropical and equatorial Atlantic, based on MOCNESS plankton net samples (their Table 5: day sampling, 270 ± 90 ind. per 1000 m^3^; night sampling, 227 ± 136 ind. per 1000 m^3^), this likely reflects the low catchability of faster-swimming organisms by the Multinet, which tends to undersample taxa capable of net avoidance.

The depth distribution of transforming to adult *Cyclothone* in the Atlantic is known to vary among species and may extend well below the mesopelagic zone for some species (Sarmiento-Lezcano *et al.,* 2022). In our study, however, sampling was limited to 800 m even though station depths reached 3000 m. *Cyclothone* remained within the mesopelagic domain both day and night, confirming its non-migratory role as a resident component of the deep scattering layers, consistent with observations from the Atlantic (Olivar *et al.,* 2012, 2018; Dove *et al.,* 2021; Sarmiento-Lezcano *et al.,* 2022) and other oceanic regions worldwide (e.g. Sassa *et al.,* 2007; Olivar *et al.,* 2016, 2022). In contrast, transforming/juvenile stages of myctophids, phosichthyids and sternoptychids tended to ascend at night, whereas no pattern was resolved for stomiids; the latter was only incidentally detected in a single photoperiod (night-only), DVM estimation. The observed nocturnal upward migration is consistent with previous studies reporting taxon-specific patterns linked to ontogenetic changes in morphology and physiology (Olivar *et al.,* 2018; Dove *et al.,* 2021). Because WMD and DVM estimates were derived from relatively coarse 200 m-thick depth strata, these values should be interpreted with caution, as this vertical resolution may smooth out finer-scale vertical structure and vertical movements. In this context, it is important to recall that only four complete day-and-night sampling cycles could be carried out over the course of the survey. Consequently, the results should be interpreted with appropriate caution due to the limited sample size.

Beyond physiological and ontogenetic factors, environmental gradients also influenced the vertical distribution of mesopelagic fishes. In our study area, an Oxygen Minimum Zone (OMZ) varied along the transect occurring above ~ 400 m at station 5 and generally below ~ 600–700 m at the deeper stations with local minima near ~ 2 mL L^−1^. Such values fall within the range of hypoxia defined by Díaz and Rosenberg (2008) and are close to levels reported to induce metabolic stress in larval fishes (Ekau *et al.,* 2010). Nevertheless, several transforming to adult specimens of various taxa were recorded near or within the upper limit of this layer, implying variable tolerance to oxygen depletion. The occurrence of mesopelagic families in these deeper waters underscores their documented capacity to thrive under low-oxygen conditions (Olivar *et al.,* 2017; Sánchez-Velasco *et al.,* 2019). In particular, the predominance of *Cyclothone* aligns with previously observed patterns, as species of this genus are known for their remarkable ability to withstand low-oxygen environments (e.g. Olivar *et al.,* 2017). This tolerance could be attributed to physiological and behavioral adaptations such as efficient oxygen extraction, reduced metabolic demands, and minimal locomotor activity, which together enable them to persist and feed in moderate hypoxic waters.

Multinet biomass showed a depth-related pattern, characterized by higher values in the upper 0–100 m layer and a progressive decline with depth, following the general pattern expected for pelagic ecosystems. For instance, Hernández-León *et al.* (2019a) reported a similar pattern from 0–800 m in the oceanic Canary Current (station 12, closest to our transect), characterized by a pronounced subsurface maximum at 0–100 m with decreasing biomass below this layer both day and night. However, the most inshore station sampled by the Multinet in our study (station 5) displayed notably high biomass even in the deepest layers, suggesting the influence of local processes or environmental conditions that enhance secondary production or promote aggregation at depth (Hernández-León *et al.,* 2020; Sarmiento-Lezcano *et al.,* 2022). Consistently, the relative contribution of the > 2000 μm fraction and transforming/juvenile fish stages increased with depth and shoaled at night, indicating diel vertical migration within these communities. Such patterns contribute to carbon transfer at depth through complementary active and passive fluxes (Steinberg and Landry, 2017).

Alongside transforming/juvenile stages of other mesopelagic fishes and zooplankton, *Cyclothone* persistently occupied the deep layers (400–800 m) with substantial biomass. However, the total biomass of the genus was likely underestimated by our Multinet catches, since larger individuals are typically captured with alternative gears such as midwater (pelagic) trawls. As reported by Agersted *et al.* (2021), up to 40% of *Cyclothone* biomass may be represented in these alternative catches, further reinforcing its role in mesopelagic carbon remineralization through respiration, excretion and trophic interactions (Sarmiento-Lezcano *et al.,* 2022). Within the 600–800 m stratum, dry weight ranged from 42.3 to 1017.4 mg per 1000 m^3^ (≈16.9–407.0 mg C per 1000 m^3^ assuming 40% carbon; Omori and Ikeda, 1984; Escribano *et al.,* 2007), values broadly comparable to those reported by Sarmiento-Lezcano *et al.* (2022) for the 400–800 m layer where *Cyclothone* biomass covaried with net primary production. As already observed in other oceanic studies (e.g. Dove *et al.,* 2021; Olivar et al., 2022), the 1 m^2^ Multinet collected several mesopelagic taxa in addition to Cyclothone, including micronektonic organisms. However, we acknowledge that larger and faster–swimming specimens are likely under-sampled because they can avoid plankton nets, and our estimates should therefore be interpreted conservatively. Despite this limitation, the dry–weight calculations from our catches suggest that these non–Cyclothone taxa can contribute substantially to total biomass in the deeper layers (400–800 m), highlighting their potential relevance for carbon cycling through respiration and remineralization processes.

Our study provides the first longitudinal (inshore–offshore) study combining vertical patterns of transforming/juvenile fish and larval fish assemblages in the CCLME. The transect explicitly spans the coastal upwelling gradient from the nearshore upwelling zone to stratified offshore waters. Elevated biomass in the upper 0–100 m layer and the strong contribution of large zooplankton fraction and transforming/juvenile fish stages at 400–800 m layer highlight the role of vertical and spatial heterogeneity in carbon cycling. Within the 400–800 m strata, transforming to adult stages of *Cyclothone* dominated the mesopelagic assemblage sampled by the Multinet, while other mesopelagic taxa were less abundant and showed shallower nocturnal distributions. These findings establish a baseline for understanding the vertical organization of early life stages and mesopelagic community dynamics in this major eastern boundary upwelling system. Building on this snapshot, and in line with contrasts in zooplankton biomass between the south-of-Canary-Islands and Cape Blanc transects reported by Hernández-León et al. (2019b) and latitudinal shifts reported as well by Goliat et al., 2025 along the CCLME; a comparative framework encompassing multiple latitudes appears essential to sort out how upwelling intensity, mesoscale activity, and OMZ structure shape transforming to adult stages across the CCLME.

## CONCLUSIONS

The upwelling regime of the Canary Current Large Marine Ecosystem (CCLME) generates pronounced horizontal and vertical gradients in physical conditions, which are reflected in the structure of larval and transforming/juvenile stage fish assemblages. This study further displays that transforming to adult stages of *Cyclothone* residing mainly between 400 and 800 m depth, represent a major component of mesopelagic biomass and likely play a key role in carbon flow through respiration and remineralization processes, as suggested by previous studies. Transforming/juvenile stages of other mesopelagic taxa also contribute substantially to this carbon flux, not only through their biomass but also via diel vertical migration, which facilitates active carbon transport across depth layers. Together, these findings highlight the strong coupling between the physical structure of the upwelling system and the biological processes within the CCLME.

## Supplementary Material

fbag030_Supplementary_materials

## Data Availability

The data were collected within the framework of the EAF-Nansen Programme and can be requested through the Food and Agriculture Organization of the United Nations (FAO) webpage (https://www.fao.org/in-action/eaf-nansen/data/data-access/en).
