# Supplementary material for: Spatial distribution of early life stages of fish along horizontal and vertical gradients in the canary current large marine ecosystem
Source: J Plankton Res. 2026 May 20;48(3):fbag030. doi: 10.1093/plankt/fbag030 (PMC13189272; doi:10.1093/plankt/fbag030)
Supplement: fbag030_Supplementary_materials [file fbag030_supplementary_materials.docx]

# SUPPLEMENTARY DATA

# TABLES

| ***Table S1.*** *Average abundance (ind. m^-2^) from Bongo net samples and contribution (%) from the SIMPER analysis on the larval fish taxa responsible for >70% of similarity within the station groups identified by cluster analysis. The standard error is shown in parentheses. The blue colour gradient indicates high and low contribution. Absence of taxa and negligible % contribution are* indicated as “-“. | | | | |
| --- | --- | --- | --- | --- |
| **Taxa** | **Average density** | | **Contribution** | |
|  | **A** | **B** | **A** | **B** |
| *Sardina pilchardus* | 51.79 (3.94) | - | 80.93 | - |
| *Cyclothone* spp. | - | 0.87 (2.50) | - | 27.29 |
| *Maurolicus* spp. | 1.11 (0.60) | 0.72 (0.67) | 5.01 | 10.11 |
| *Vinciguerria nimbaria* | - | 0.91 (0.73) | - | 9.53 |
| *Diaphus* spp. | 0.50 | 0.78 (0.71) | - | 9.06 |
| *Hygophum proximum* | - | 0.97 (0.43) | - | 0.43 |
| *Lampanyctus* spp. | 0.40 | 1.03 (0.71) | - | 5.88 |
| *Hygophum reinhardtii* | - | 0.22 (0.48) | - | 4.58 |

| ***Table S2.*** *Day and night weighted mean depth (WMD) and diel vertical migration (DVM) of transforming/juvenile mesopelagic stages* *(including Cyclothone “transforming to adult”) at each sampling station.* | | | | | | |
| --- | --- | --- | --- | --- | --- | --- |
| **Stage** | **Family** | **Taxa** | **Station** | **WMD** | | **DVM** |
|  |  |  |  | **Day** | **Night** |  |
| Transforming | Sternoptychidae | *Argyropelecus hemigymnus* | 7 | 550.50 | 548.50 | -2.00 |
|  |  |  | 9 | 421.40 | 252.20 | -169.20 |
|  |  | *Maurolicus* spp. | 11 | 302.00 | 301.00 | -1.00 |
|  | Myctophidae | Myctophidae spp. | 9 | 699.50 | 505.36 | -194.14 |
|  |  |  | 11 | 683.50 | 499.00 | -184.50 |
|  | Phosychthyidae | *Vinciguerria* spp. | 7 | 476.78 | 51.50 | -425.28 |
|  | Gonostomatidae | *Cyclothone* spp. | 7 | 660.23 | 602.40 | -57.83 |
|  |  |  | 9 | 532.16 | 555.23 | 23.08 |
|  |  |  | 11 | 523.28 | 544.26 | 20.98 |
| Early | Gonostomatidae | *Cyclothone* spp. | 7 | 52.00 | 51.50 | -0.50 |
|  |  |  | 11 | 51.50 | 149.00 | 97.50 |

# FIGURES


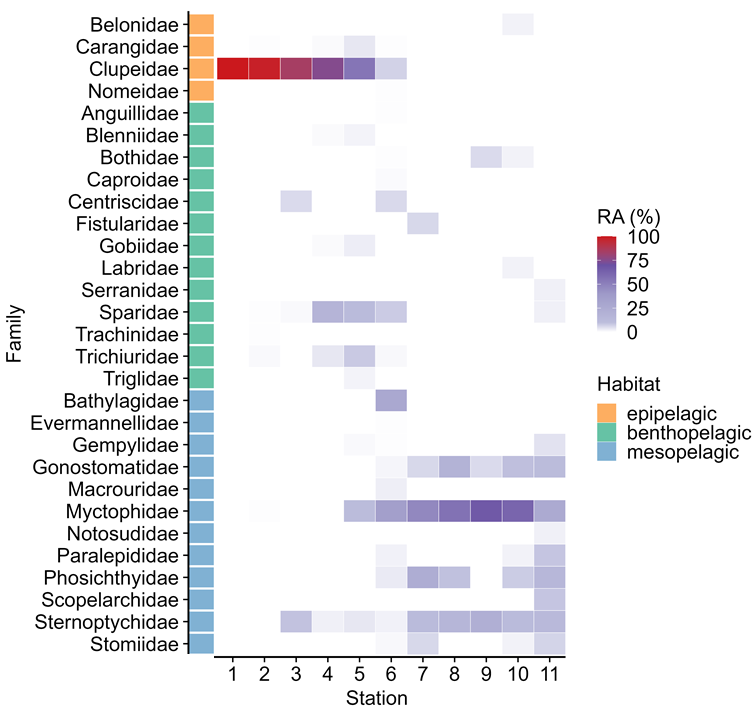


## **Figure S1**

Heat map showing the relative abundance (RA, %) of fish larvae on family level collected with the Bongo net along the transect. Color intensity (white – purple - red) indicates the proportional contribution of each family at each station. Color sidebar represents the adult habitat types (epipelagic, benthopelagic, mesopelagic).


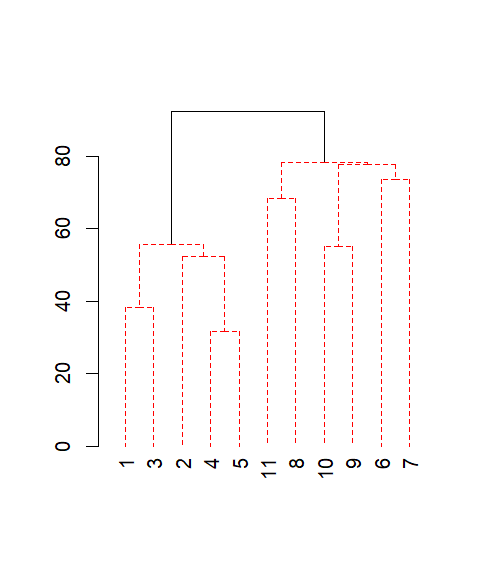


## **Figure S2**

Result of Similarity Profile analysis (SIMPROF) based on square-root transformed larval abundances using Bray–Curtis dissimilarity and group-average linkage (UPGMA) from Bongo net samples. Two significant (α = 0.05) groups (A: station 1–5; B: station 6–11) were identified at 79% dissimilarity (dashed lines).


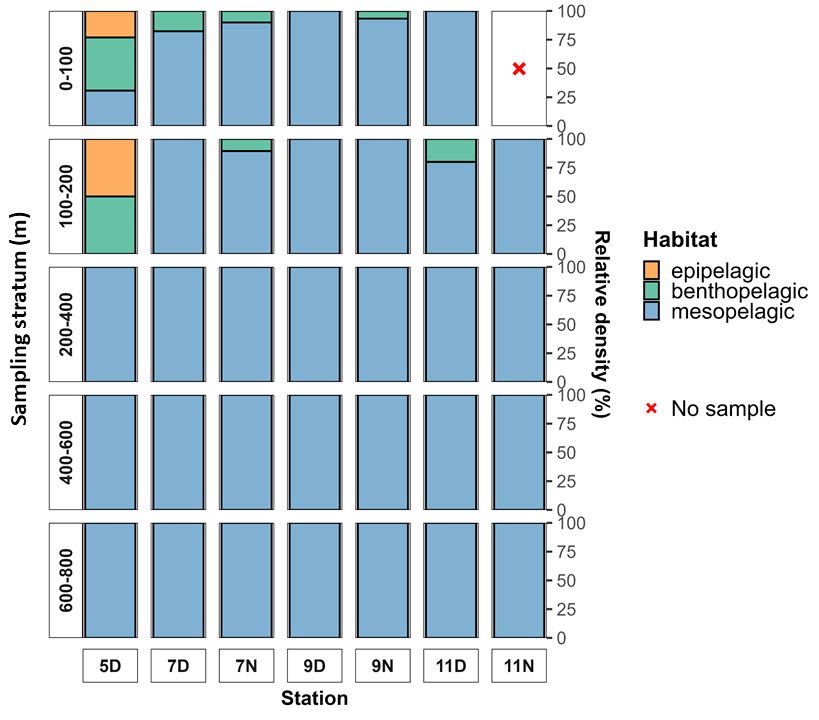


## **Figure S3**

Relative density (%) of fish larvae classified by adult habitat (epipelagic, mesopelagic, benthopelagic), across depth strata (0–100, 100–200, 200–400, 400–600, and 600–800 m) collected with the Multinet along the transect. Light conditions during sampling are indicated for day (D) and night (N) next to the station number. The red cross denotes the station and stratum where no sample was obtained.

##
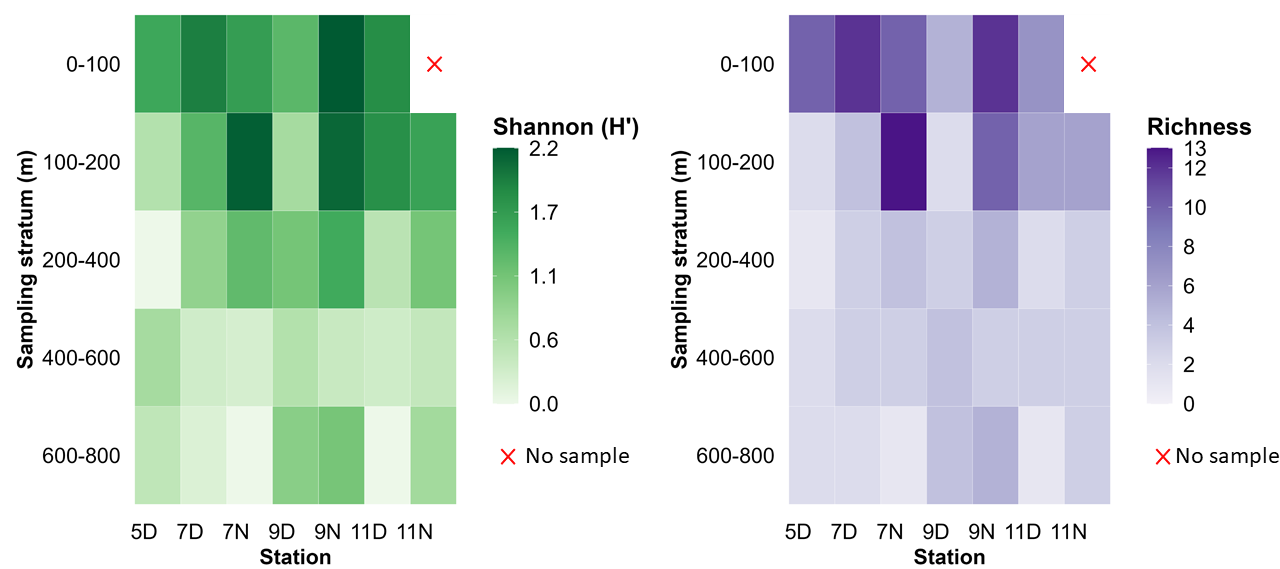
Figure S4

Heatmaps of the larval fish diversity with Shannon (H’) diversity index (left) and richness (right) across stations and depth strata (0–100, 100–200, 200–400, 400–600, and 600–800 m) from Multinet samples. Light conditions during sampling are indicated by day (D) and night (N) next to the station number. Color gradients indicate diversity indices according to the respective scales. The red cross denotes the station and stratum where no sample was obtained.


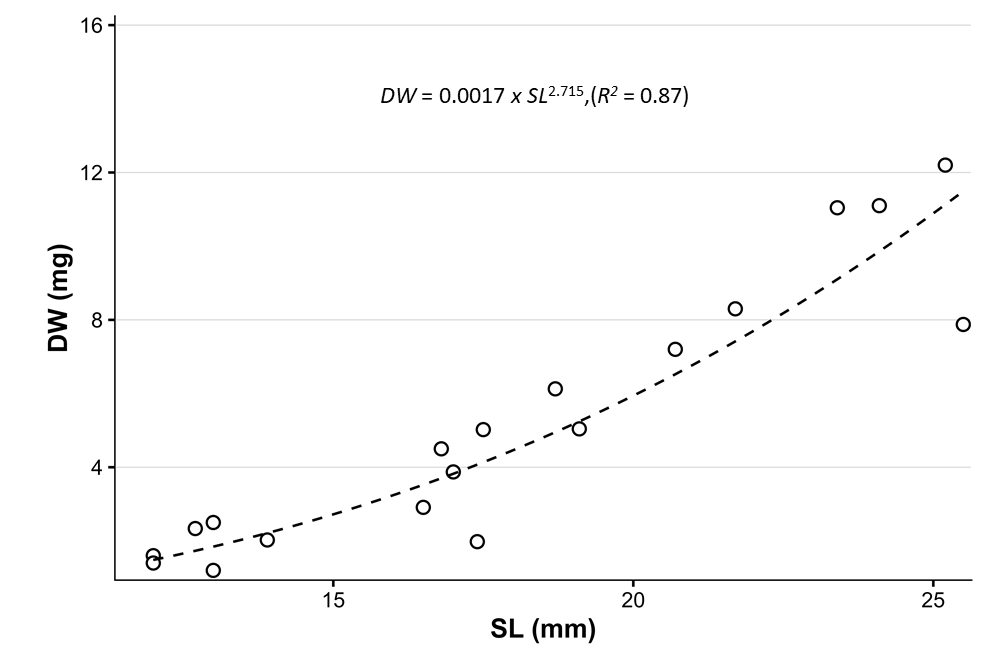


## Figure S5

Length–dry weight regression for transforming stages of *Cyclothone* spp. Values represent mean measurements per sample (19 samples; *n* = 282 *Cyclothone* individuals measured across all multinet samples). SL: standard length (mm); DW: dry weight (mg).
